# Human neutrophil lipocalin, procalcitonin, c-reactive protein, and leucocyte count for prediction of bacterial sepsis in emergency department patients

**DOI:** 10.1186/s13049-025-01429-9

**Published:** 2025-07-01

**Authors:** Jonathan Benhamou, Ricardo Nieves-Ortega, Christian H. Nickel, Alina Lampart, Tobias Kuster, Gianmarco M. Balestra, Christiane Rosin, Christoph Becker, Kriemhild Lippay, Roland Bingisser

**Affiliations:** 1 Emergency Department, University Hospital Basel, Petersgraben 2, Basel, 4031 Switzerland; 2 Department of Intensive Care, University Hospital Basel, Petersgraben 4, Basel, 4031 Switzerland

**Keywords:** Emergency medicine, Bacterial sepsis, Biomarkers, Human neutrophil Lipocalin, C-reactive protein, Procalcitonin

## Abstract

**Background:**

Delayed identification of bacterial sepsis undermines the initiation of antibiotic and other time-sensitive treatments in the emergency department (ED). We aimed to investigate the performance of human-neutrophil lipocalin (HNL), procalcitonin (PCT), C-reactive protein (CRP), and leucocyte count in conjunction with clinical scores for the early prediction of bacterial sepsis.

**Methods:**

Patients presenting to the emergency department (ED) with a suspected infection and a national early warning score (NEWS) ≥ 2 at triage were screened for eligibility. The study biomarkers were measured at ED presentation. The primary outcome was bacterial sepsis, defined as an acute bacterial infection and an increase of ≥ 2 points in the sequential organ failure assessment (SOFA) score (Sepsis-3 criteria). The diagnostic accuracy of the biomarkers for bacterial sepsis was calculated using receiver operating curve (ROC) analysis and its area under the curve (AUC) with 95% confidence intervals (CI).

**Results:**

In total, we included 421 patients, of whom 155 (36.8%) had bacterial sepsis. For the prediction of bacterial sepsis, PCT outperformed the other biomarkers with an AUC (95% CI) of 0.77 (0.72–0.82), compared to HNL 0.72 (0.67–0.77), CRP 0.71 (0.66–0.76), and leucocyte count 0.64 (0.59–0.70). A combination of serum HNL with NEWS and SOFA at presentation had the best predictive value for bacterial sepsis at 48 h after ED presentation (AUC 0.83).

**Conclusion:**

Our findings do not support the routine use of serum HNL as a tool in the prediction of bacterial sepsis in patients presenting to the ED. A combination of biomarkers (serum HNL or CRP plus leucocytes) with NEWS and SOFA at presentation outperformed inflammatory biomarkers used individually in the prediction of bacterial sepsis.

**Supplementary Information:**

The online version contains supplementary material available at 10.1186/s13049-025-01429-9.

## Background

Early detection of bacterial sepsis is one of the major challenges in emergency medicine, as time to antibiotic treatment is associated with mortality [1, 2]. Many strategies have been used to improve timely initiation of antibiotics while preventing overuse, including clinical scores, biomarkers, and various combinations [3–5]. To date, no ideal biomarker has been identified for early detection of sepsis [6]. C-reactive protein (CRP) and procalcitonin (PCT) have been studied extensively regarding the diagnosis and prognosis of sepsis and compared to the performance of other biomarkers [7–11]. CRP has shown poor specificity for sepsis [12]. PCT may have the best performance for the diagnosis and prognosis of sepsis [13]. However, a meta-analysis indicated a limited value of PCT for the diagnosis of sepsis [14], while the 2021 Surviving Sepsis Campaign guidelines even discouraged the use of PCT to assist in the diagnosis and initial management of sepsis [15].

Human neutrophil lipocalin (HNL), also known as neutrophil gelatinase-associated lipocalin (NGAL), is a protein released by neutrophils during infection and inflammation [16]. Elevated levels of HNL have been shown to be associated with sepsis in critically ill patients, independent of the level of renal dysfunction [17]. HNL outperformed CRP and PCT in the distinction between viral and bacterial infections in an observational study in hospitalized patients with signs and symptoms of acute infection [18]. HNL may also be superior to CRP and PCT in detecting bacterial infection in critically ill sepsis patients [19].

Only few studies have focused on the identification of bacterial sepsis as the primary outcome in patients presenting to the emergency department (ED), which is crucial for early rule-in or rule-out strategies and antibiotic stewardship in the ED, and the role of HNL in this setting is unknown. We hypothesized that HNL is a useful biomarker for predicting bacterial sepsis within 48 h of ED presentation in patients with suspected infection presenting to the ED. Therefore, we carried out a prospective observational study to assess the diagnostic performance of three easily available inflammatory biomarkers (PCT, CRP and leukocyte count) as well as HNL, to predict sepsis in ED patients with suspected infection and a national early warning score (NEWS) of ≥ 2 at triage [20].

## Methods

### Study design and patient population

Prospective observational cohort study with a 30-day follow-up in a single Swiss tertiary ED with a yearly census of over 55,000 patients. We included adult patients, aged 18 years and older, presenting to the ED with (1) a suspicion of infection (signs and symptoms compatible with the most common causes of sepsis: pneumonia, urinary tract infection, intraabdominal infection, blood-stream infection (including catheter-related infection), skin infection and meningitis) and (2) a NEWS ≥ 2 at triage (excluding patients with minimal changes of vital signs). Patients were excluded if they were younger than 18 years of age, had known pregnancy at the time of inclusion or were on antibiotic therapy initiated > 24 h before presentation to the ED. Patient recruitment took place between May 5, 2017 and May 31, 2018. The local ethics committee approved the study protocol (2017-00092, www.eknz.ch). Participants provided written informed consent. For participants unable to consent, an emergency physician unrelated to the study provided written confirmation stating that the patient’s interest was safeguarded before inclusion. This was followed by surrogate written confirmation of consent from a proxy as soon as possible. We report the study in compliance with the STROBE (Strengthening the Reporting of Observational Studies in Epidemiology) guidelines [21]. The study was conducted in accordance with the Declarations of Helsinki.

### Study protocol

Triage liaison physicians [22] routinely triaged patients using the German version of the Emergency Severity Index (ESI) [23] and recorded a complete set of vital signs and the working hypothesis in the electronic health record (EHR). The quick SOFA score (qSOFA) was calculated from the recorded vital signs. A study team consisting of medically trained staff screened patients after triage from 8 am to 8 pm. The study team approached eligible patients and asked for consent to participate. Diagnostic tests, treatment, and disposition were carried out at the treating physician’s discretion as clinically indicated.

A blood sample was drawn from all participants at the time of inclusion to measure biomarkers and carry out blood gas analyses. Blood samples for all biomarkers apart from HNL were analyzed in the laboratory of the University Hospital of Basel within 30 min of collection. Differential peripheral blood counts were performed using flow cytometry. CRP was measured using a turbidimetric assay (Dade Behring, Paris, France) with a detection limit of 0.3 mg/l. PCT was measured using an electrochemiluminescence immunoassay (Brahms Diagnostica GmbH, Berlin, Germany) with a detection limit of 0.02 ng/ml. HNL samples were analyzed in a single batch analysis using frozen serum samples after inclusion of the last patient. HNL was measured in serum using a sandwich enzyme-linked immunosorbent assay (ELISA) in an external laboratory (Diagnostics Development, Uppsala, Sweden) with a detection limit of 0.039 µg/l.

Data were recorded in electronic case report forms (secuTrial^®^, InterActive Systems GmbH, Berlin, Germany). We matched the study database with the hospital’s EHR to retrieve information on demographics, diagnostic tests, treatments, and adverse outcomes. A follow up was conducted 30 days after presentation to the ED using the EHR (re-presentations to the hospital), the official civil registry, and telephone interviews with primary care providers, patients, and proxies.

### Primary outcome

The primary outcome was bacterial sepsis, defined according to the 2016 Sepsis-3 definitions [24], as an acute bacterial infection and two or more points in the sequential organ failure assessment (SOFA) score at presentation to the ED or within 48 h of presentation. Cases of co-infection with bacterial and non-bacterial pathogens were analyzed as bacterial.

We used a multi-step process to adjudicate the infection status into four groups: “acute bacterial infection”, “acute viral infection”, “other infection” (e.g., acute fungal or parasitic) and “no acute infection”. First, a study physician extracted all positive microbiological tests drawn within 48 h of presentation, including microbiological cultures, polymerase chain reaction assays, serologic and antigen detection tests. Cases with positive microbiological tests compatible with the clinical context (e.g., pneumonia with positive blood cultures for *S. pneumoniae*) were classified accordingly.

Second, the remaining cases (e.g., no positive microbiological test, or contamination suspected) were adjudicated using a modified Delphi method. A study physician blinded to the study hypothesis carried out a protocolized chart abstraction based on triage forms, vital sign curves, discharge reports, follow-up letters and autopsy reports. Three specialists in the fields of intensive care, infectious diseases, and emergency medicine, blinded to the values of the study biomarkers, independently adjudicated each case. If all three adjudicators agreed, the case was categorized accordingly. Cases with disagreements were decided by a fourth adjudicator.

Finally, we calculated the highest SOFA score within 48 h of presentation for all participants using vital signs (Glasgow Coma Scale, mean arterial pressure) and laboratory measurements (PaO2/FiO2 ratio from arterial blood gas, platelets, bilirubin, and creatinine). We assumed increases in the SOFA score to be acute unless a chronic organ dysfunction was documented in the EHR. We obtained a venous blood gas analysis from each participant at inclusion and calculated their PaO2 using a validated algorithm [25].

### Secondary outcomes

Admission to a hospital ward, length of hospital stay (LOS), admission to an intensive care unit (ICU), length of ICU stay, all-cause 7-day, and 30-day mortality were predefined secondary outcomes. We defined admission as a disposition to any ward in any acute care hospital. We defined LOS as days between presentation to the ED and discharge from the hospital. We defined admission to the ICU as an admission to an ICU during index hospitalization. We defined time to death as days between presentation and death.

### Statistical analysis

The normality of continuous variables was tested by Shapiro–Wilk tests. Continuous variables were presented as median and interquartile range (IQR) for non-normally distributed data or mean and standard deviation (SD) for normally distributed data. Categorical variables were displayed as frequencies and percentages. We compared the distribution of continuous variables using the Wilcoxon rank sum test with continuity correction, when applicable. We used Pearson’s Chi-square test with Yate’s correction, when applicable, to compare the distribution of categorical variables. We used Bonferroni corrections to adjust for multiple comparisons.

We calculated each biomarker’s receiver operating characteristic (ROC) curve and its area under the curve (AUC) for the outcome of bacterial sepsis with 95% confidence intervals (CI). We carried out one-to-one AUC comparisons using DeLong’s test with a 5% alpha-level (two sided). Sensitivity, specificity, positive likelihood ratio (+ LR) and negative likelihood ratio (-LR) were calculated for specific biomarker cut-offs. Furthermore, a multivariate analysis was performed using a stepwise regression to create a model which best predicted bacterial sepsis, which we then compared to the biomarkers’ performances. We estimated a sample size of 403 patients to reach a statistical power of 80% based on published data about the mean concentration of HNL in patients with bacterial infection [18] and assuming an incidence of bacterial infection of 33% in our cohort. The level of significance was two sided, with a significance level of α = 0.05. A complete case analysis was performed as missing data were missing completely at random.

The statistical analysis was conducted using the R software environment for statistical computing (R version 4.3.1).

## Results

### Patient population

**Fig. 1:**
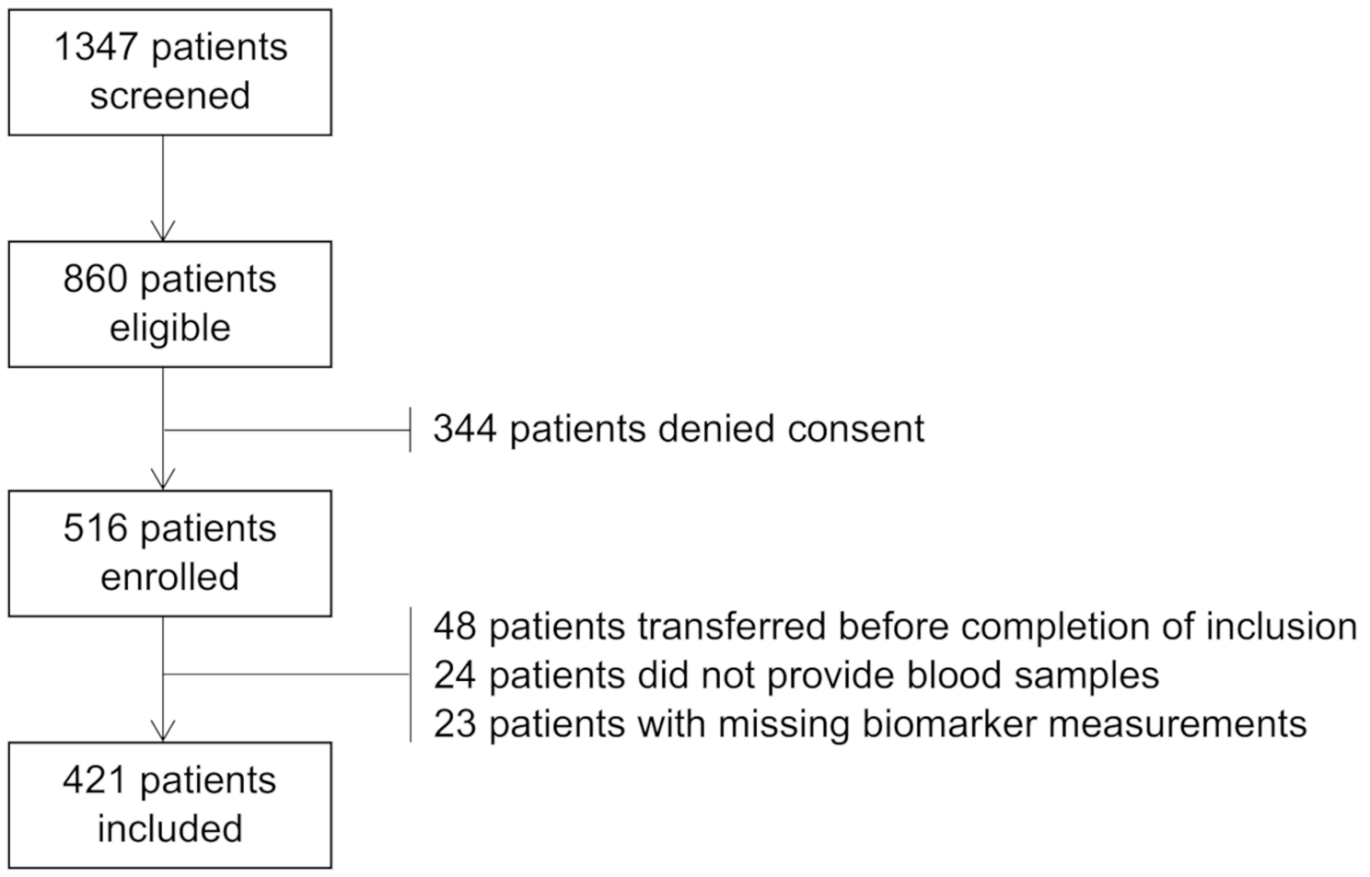
Inclusion flowchart

We screened 1347 patients during the study period, of whom 860 were eligible and 421 were included in the final cohort (Fig. 1). Median age was 68 years, 183 (43.5%) participants were female and the median NEWS at the time of presentation was 5 (IQR 3–7). Most patients were assigned an ESI of 2 (56.8%) or 3 (40.9%). Three hundred sixty-four (86.5%) patients were admitted to the hospital, 35 (8.3%) patients were admitted to the ICU, and 30 (7.1%) patients died within 30 days. SOFA scores were available for all patients. Three (0.7%) patients were lost to follow-up after 30 days (Table 1). Biomarker measurements were missing in 19 (4.3%) cases for PCT and in 4 (0.9%) cases for HNL.

**Table 1:** Baseline population characteristics at ED presentation

	Overall (*N* = 421)
Age in years, median (IQR)	68 (51–80)
Female sex, *n* (%)	183 (43.5)
ESI, *n* (%)	
1	6 (1.4)
2	239 (56.8)
3	172 (40.8)
4	4 (1.0)
NEWS, median (IQR)	5.0 (3.0–7.0)
Suspected infection focus	
Respiratory, *n* (%)	231 (54.9)
Urinary, *n* (%)	66 (15.7)
Abdominal, *n* (%)	58 (13.8)
Skin, *n* (%)	25 (5.9)
Central nervous system, *n* (%)	12 (2.9)
Bloodstream, *n* (%)	3 (0.7)
Other, *n* (%)	14 (3.3)
Unclear focus, *n* (%)	61 (14.5)
Biomarkers	
Serum PCT (ng/ml), median (IQR)	0.2 (0.1–0.5)
Serum CRP (mg/l), median (IQR)	56.9 (21.2–157.9)
Serum HNL (µg/l), median (IQR)	191.8 (130.3–271.7)
Leucocyte count (x10^9^/l), median (IQR)	10.7 (7.7–14.1)
Outcomes	
Hospital admission, *n* (%)	364 (86.5)
Length of hospital stay in days, median (IQR)	8.0 (5.0–14.0)
Admission to ICU, *n* (%)	35 (8.3)
Length of ICU stay in days, median (IQR)	1.8 (0.9–4.5)
7-day mortality, *n* (%)	28 (6.7)
30-day mortality, *n* (%)	30 (7.1)
Lost to follow-up, *n* (%)	3 (0.7)

IQR = interquartile range; n = number; ESI = Emergency Severity Index; NEWS = National Early Warning Score; CRP = C-reactive protein; PCT = procalcitonin; HNL = human neutrophil lipocalin; ICU = intensive care unit

### Outcomes

Overall, 69 (16.4%) patients were found to have no acute infection, 241 (57.2%) had a bacterial infection, 108 (25.7%) a viral infection and 3 (0.7%) an infection caused by other pathogens. Twenty-five patients had a bacterial and viral co-infection and were subsequently categorized as bacterial. The infection status was adjudicated by microbiological evidence in 192 (45.6%) cases, by expert consensus in 167 (39.7%) cases and by a fourth referee in 62 (14.7%) of all cases.

Two hundred and three (48.2%) patients had an increase of ≥ 2 points in SOFA scores within 48 h of presentation. Of those, 155 patients (36.8%) had an acute bacterial infection, categorizing them as having bacterial sepsis, while 48 patients (11.4%) had an acute viral infection and were categorized as having viral sepsis. Conversely, 218 patients (51.8%) did not experience an increase of ≥ 2 points in their SOFA scores and were classified as not having sepsis. Patients with bacterial sepsis had a higher hospital admission rate, longer LOS and higher 7-day and 30-day mortality than patients with viral sepsis and patients without sepsis. A little over 10% of patients with viral and bacterial sepsis were admitted to ICU (versus 5.5% in patients with no sepsis) with patients with viral sepsis having the longest ICU LOS (Table 2).

**Table 2:** Characteristics and outcomes according to sepsis pathogen

	No sepsis (*N* = 218)	Bacterial sepsis (*N* = 155)	Viral sepsis (*N* = 48)
Age in years, median (IQR)	63 (43–76)	77 (65–85)	63 (56–75)
Female sex, *n* (%)	99 (45.4)	61 (39.4)	23 (47.9)
NEWS, median (IQR)	4.0 (3.0–6.0)	7.0 (4.0–9.0)	6.0 (4.0–7.0)
SOFA at presentation, median (IQR)	0.0 (0.0–0.0)	2.0 (2.0–3.0)	2.0 (2.0–3.0)
SOFA after 48 h, median (IQR)	1.0 (0.0–1.0)	3.0 (2.0–4.0)	3.0 (2.0–4.0)
Outcomes			
Hospital admission, *n* (%)	170 (78.0)	149 (96.1)	45 (93.8)
Length of hospital stay in days, median (IQR)	7.0 (4.0–13.0)	9.0 (6.0–19.0)	8.0 (4.0–14.0)
Admission to ICU, *n* (%)	12 (5.5)	17 (11.0)	6 (12.5)
Length of ICU stay in days, median (IQR)	0.9 (0.7–2.0)	2.0 (0.9–6.0)	3.4 (2.3–8.2)
7-day mortality, *n* (%)	4 (1.8)	22 (14.2)	2 (4.2)
30-day mortality, *n* (%)	4 (1.8)	24 (15.5)	2 (4.2)

IQR = interquartile range; n = number; ESI = Emergency Severity Index; NEWS = National Early Warning Score; SOFA = Sequential Organ Failure Assessment; CRP = C-reactive protein; PCT = procalcitonin; HNL = human neutrophil lipocalin; LOS = length of stay; ICU = intensive care unit

**Fig. 2:**
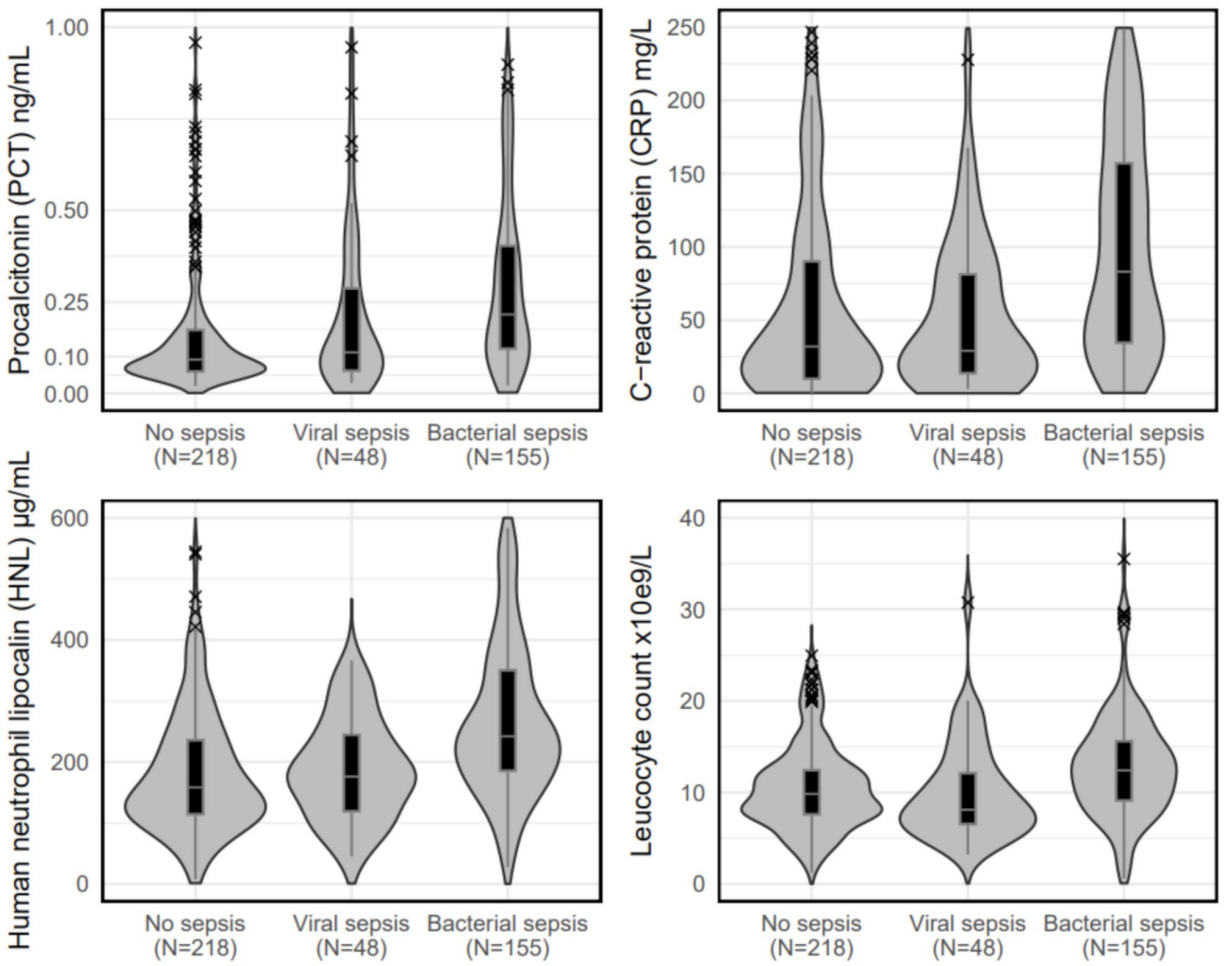
Biomarker levels by sepsis pathogen Patients with bacterial sepsis (*n* = 155), viral sepsis (*n* = 48) and no sepsis (*n* = 218) are shown. The interquartile range is represented by box plots inside the violin plots

When compared with patients without sepsis, patients with bacterial sepsis were older (*p* < 0.001), had higher NEWS (*p* < 0.001) and SOFA (*p* < 0.001) at presentation, and higher PCT (*p* < 0.001), higher serum HNL (*p* < 0.001), higher CRP (*p* < 0.001) levels, and higher leucocyte counts (*p* < 0.001); they also had significantly worse outcomes with higher hospitalization rates (*p* < 0.001), longer LOS (*p* < 0.001), and higher all-cause mortality (*p* < 0.001). When compared with patients with viral sepsis, patients with bacterial sepsis were older (*p* < 0.001), had higher PCT (*p* < 0.001), higher serum HNL (*p* < 0.001), higher CRP (*p* < 0.001) and higher leucocyte count (*p* < 0.004); they also had higher 7-day and 30-day mortality (*p* < 0.001) but no difference in the other measured outcomes. Figure 2 shows the distribution of the study biomarkers according to the sepsis pathogen.

### Biomarker and risk assessment tool performance to predict bacterial sepsis

**Fig. 3:**
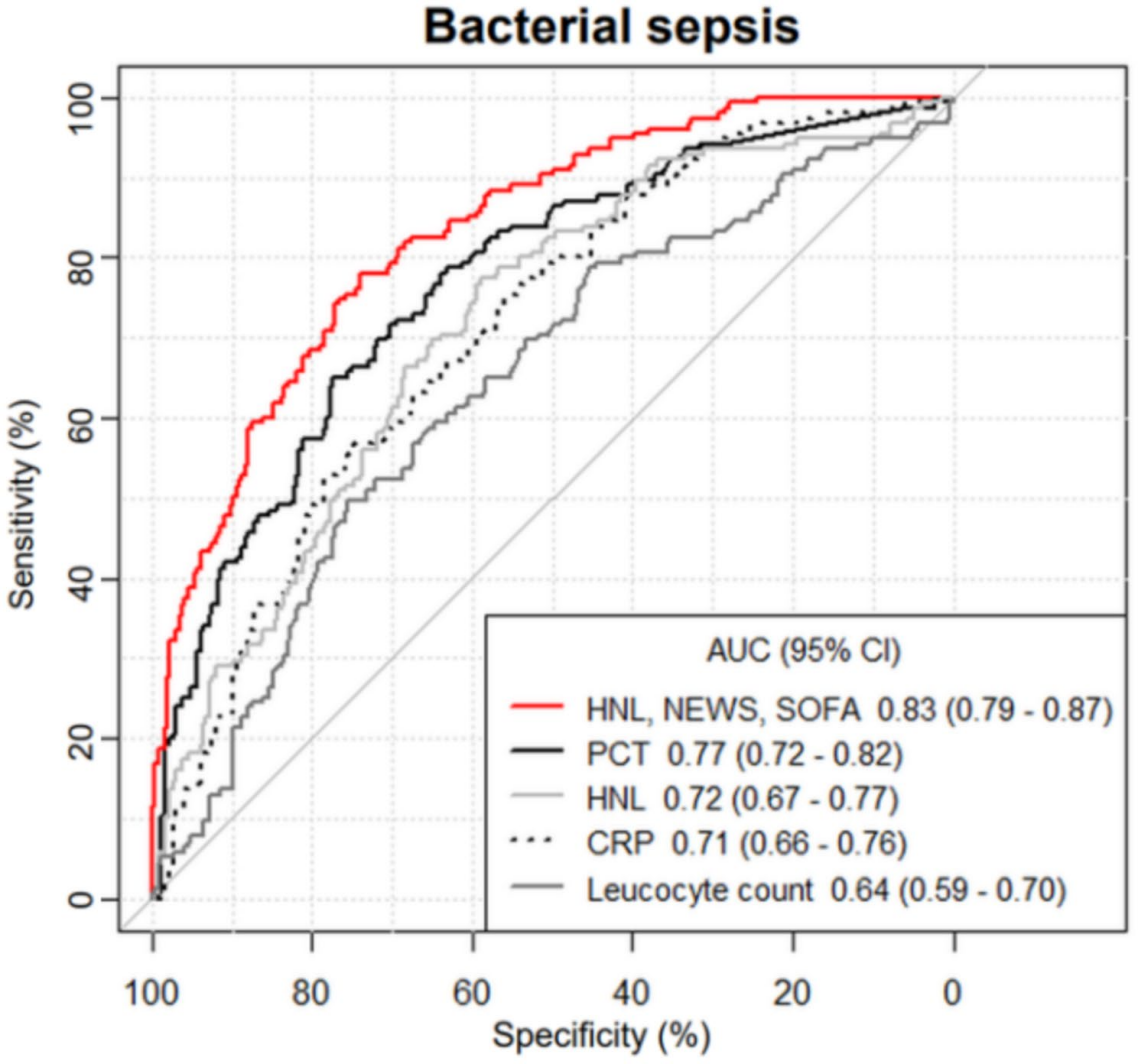
ROC analysis of a model including HNL + NEWS + SOFA, and individual biomarkers to predict bacterial sepsis ROC = receiver operating characteristic; AUC = area under the curve; CI = confidence interval; PCT = procalcitonin; CRP = C-reactive protein; HNL = human neutrophil lipocalin

The ROC analysis of the study biomarkers for the outcome of bacterial sepsis showed the following AUC (95%CI): serum HNL 0.72 (0.67–0.77), PCT 0.77 (0.72–0.82), CRP 0.71 (0.66–0.76), and leucocyte count 0.64 (0.59–0.70) (Fig. 3). For the prediction of bacterial sepsis, PCT outperformed serum HNL (*p* = 0.049), CRP (*p* = 0.033) and leucocyte count (*p* < 0.001). Serum HNL (*p* = 0.007) and CRP (*p* = 0.033) both outperformed leucocyte count. The ROC analysis of assessment scores for the outcome of bacterial sepsis showed the following AUC (95%CI): quick SOFA (qSOFA) 0.69 (0.64–0.74), NEWS 0.72 (0.67–0.77), and SOFA at presentation to the ED 0.83 (0.80–0.87). The sensitivity analysis including patients with high-certainty infection status only (i.e., patients with either microbiologically confirmed infection or expert consensus, *n* = 359) showed comparable results.

The model created using a stepwise multivariate regression which best predicted bacterial sepsis included serum HNL, and NEWS and SOFA at presentation. The ROC analysis of this model had an AUC (95% CI) of 0.83 (0.79–0.87) and outperformed PCT (*p* = 0.011) in predicting bacterial sepsis (Fig. 3). According to Youden’s Index (YI), at the optimal threshold this model had a sensitivity of 78.1%, specificity of 74.1%, +LR of 3.01, and -LR of 0.29.

A model including CRP, leucocyte count, NEWS and SOFA at presentation had a similar AUC (95% CI) of 0.82 (0.78–0.86) (*p* = 0.17) and performed better than a model only including CRP, leucocyte count and NEWS (AUC 0.77, *p* = 0.001). According to YI, at the optimal threshold this model had a sensitivity of 73.5%, specificity of 76.7%, +LR of 3.16, and -LR of 0.34 (Figure S1). A model containing HNL, and NEWS and qSOFA at presentation had an AUC of 0.79, while a model containing CRP, leucocyte count, and NEWS and qSOFA at presentation had an AUC of 0.78, with both being outperformed by abovementioned models (*p* = 0.004, *p* = 0.001).

According to YI, the optimal cut-offs for prediction of bacterial sepsis were 101 mg/l for CRP (sensitivity 56.8%, specificity 74.8%, +LR 2.25, -LR 0.58), 0.27 ng/ml for PCT (sensitivity 65.2%, specificity 77.4%, +LR 2.89, -LR 0.45), 182 µg/ml for HNL (sensitivity 77.4%, specificity 59.0%, +LR 1.89, -LR 0.38), and 12.6 × 10^9^/l for leucocyte count (sensitivity 49.7%, specificity 75.6%, +LR 2.03, -LR 0.67). A CRP cut-off of 10 mg/l had a sensitivity of 96.8% and a specificity of 21.4% (+ LR 1.25, -LR 0.14). A PCT cut-off of 0.1 ng/ml had a sensitivity of 86.4% and a specificity of 48.9% (+ LR 1.71, -LR 0.26); whereas a cut-off of 0.5 ng/ml had a sensitivity of 47.1% and a specificity of 87.2% (+ LR 3.72, -LR 0.61) (Table 3).

**Table 3:** Performance of biomarker cut-offs to identify bacterial sepsis

Biomarker	Cut-off	Sensitivity, % (95%CI)	Specificity, % (95%CI)	+LR (95%CI)	-LR (95%CI)
CRP	10 mg/l	96.8 (93.5–98.5)	21.4 (17.4–25.9)	1.25 (1.12–1.39)	0.14 (0.08–0.24)
	50 mg/l	74.2 (69.6–78.3)	56.0 (50.8–61.0)	1.70 (1.35–2.14)	0.45 (0.37–0.56)
	101 mg/l (YI)	56.8 (52.0-61.5)	74.8 (70.2–78.9)	2.25 (1.78–2.84)	0.58 (0.49–0.68)
PCT	0.1 ng/ml	86.4 (82.8–89.3)	48.9 (43.9–53.9)	1.71 (1.38–2.12)	0.26 (0.21–0.32)
	0.27 ng/ml (YI)	65.2 (60.5–69.6)	77.4 (72.7–81.6)	2.89 (2.29–3.65)	0.45 (0.37–0.54)
	0.5 ng/ml	47.1 (42.4–51.9)	87.2 (83.9–89.9)	3.72 (2.93–4.72)	0.61 (0.52–0.71)
HNL	182 µg/ml (YI)	77.4 (73.2–81.1)	59.0 (53.8–64.0)	1.89 (1.50–2.37)	0.38 (0.30–0.49)
Leucocyte count	10 × 10^9^/l	69.0 (64.3–73.3)	53.4 (48.3–58.4)	1.43 (1.15–1.78)	0.61 (0.50–0.74)
	12.6 × 10^9^/l (YI)	49.7 (45.0-54.4)	75.6 (71.0-79.7)	2.09 (1.64–2.66)	0.67 (0.57–0.78)
	15 × 10^9^/l	30.3 (26.2–34.7)	83.5 (79.9–86.7)	1.72 (1.36–2.18)	0.85 (0.78–0.92)

CRP = C-reactive protein; PCT = Procalcitonin; HNL = Human Neutrophil Lipocalin; YI = Youden’s Index

The ROC analysis for the outcome of bacterial infection (with and without multiorgan dysfunction) can be found in the supplementary content (Table S1 and Figure S2).

## Discussion

This prospective study showed a high incidence of bacterial sepsis in patients presenting to the ED with suspected infection and a NEWS of ≥ 2, along with a comparably low mortality among affected patients. Combinations of biomarkers and clinical scores performed better than individual biomarkers in predicting bacterial sepsis. Specifically, a combination of serum HNL with NEWS and SOFA at presentation outperformed all individual biomarkers. A model containing CRP, leucocyte count, NEWS, and SOFA showed a comparable performance. Additionally, with a negative predictive value of 92%, CRP at a cut-off of 10 mg/l demonstrated high effectiveness in ruling out bacterial sepsis.

Data on the performance of inflammatory biomarkers in ED patients with suspected infection regarding the outcome “bacterial sepsis” are relatively rare [26]. In our study, PCT performed better than serum HNL, CRP, and leucocyte count in predicting bacterial sepsis, which is in line with other studies comparing PCT to other biomarkers [7–10, 14]. In recent years studies have focused on the diagnostic accuracy of microRNAs (small noncoding RNA) for detecting sepsis. These have shown slightly improved diagnostic accuracy when compared to serum HNL [27].

Our results are in line with recommendations made by Castillo et al. regarding the simultaneous application of risk tools to identify sepsis in the ED (one based on clinical variables such as NEWS, another based on analytical variables such as SOFA, and a biomarker) [28]. NEWS has shown superior predictive value for early deterioration in patients with sepsis in the ED than SIRS and qSOFA [29–31]. Furthermore, SOFA and changes in the SOFA score over time are useful risk stratification tools [32]. The complete SOFA outperformed qSOFA in our models, indicating that using additional parameters, such as calculated blood gases, could be beneficial. Moreover, the strong performance of a model including CRP and leucocyte count is relevant, as these biomarkers are readily available in most hospitals as compared to serum HNL. The ability of CRP to effectively rule out bacterial infection and sepsis makes it a potentially useful biomarker in the initial assessment of patients with a suspected infection, unfortunately only at very low levels. We assume that PCT in combination with clinical scores was outperformed by serum HNL and CRP + leucocyte count because the combinations integrated different aspects of sepsis without significantly overlapping in information.

Over one third of all patients with suspected infection and NEWS ≥ 2 had bacterial sepsis, as defined by the Sepsis-3 criteria, and in-hospital mortality was 14%. In-hospital mortality in patients with all types of sepsis (including viral origin) was 12%. This is considerably lower than in a previous study, in which short term mortality was over 22%. However, inclusion bias may have been involved, as half of these patients had a septic shock [2], which is not to be expected in an unselected population of ED patients with suspected infection. Furthermore, the SOFA scores and biomarker results in our study population may suggest that the disease severity was low, potentially explaining the lower mortality. Another large prospective study including ED patients with suspected infection (no vital parameter exclusion criteria) showed an in-hospital mortality of 8% [4]. A retrospective study including patients who received intravenous antibiotics, or had bacterial cultures drawn in the ED, reported a 30-day mortality of 6% [33], while a study including patients with suspected sepsis reported an in-hospital mortality of 15% [3]. These disparities between studies may be attributed to differing inclusion criteria and outcome definitions.

This study has some limitations. First, the external validity of the study is limited due to the single-center design and the non-inclusion of 51% of eligible patients. Second, only patients presenting between 8 am and 8 pm were screened for inclusion, potentially introducing a selection bias. Third, the infection status adjudication relied on expert consensus, which may introduce subjective bias, despite the use of an elaborate Delphi method.

## Conclusion

Our findings do not support the routine use of serum HNL as a tool in the prediction of bacterial sepsis in patients presenting to the ED. A combination of biomarkers (serum HNL or CRP plus leucocytes) with NEWS and SOFA at presentation outperformed inflammatory biomarkers used individually in the prediction of bacterial sepsis, which emphasizes the importance of a multifaceted approach in diagnosing sepsis.

## Electronic supplementary material

Below is the link to the electronic supplementary material.

Supplementary Material 1

## Data Availability

The dataset is not available due to ethical restrictions. The release of the dataset can be requested from the responsible ethics committee.

## Abbreviations

CRP: C-reactive protein
PCT: Procalcitonin
HNL: Human neutrophil lipocalin
NGAL: Neutrophil gelatinase-associated lipocalin
ED: Emergency department
NEWS: National early warning score
STROBE: Strengthening the reporting of observational studies in epidemiology
ESI: Emergency severity index
EHR: Electronic health record
ELISA: Enzyme-linked immunosorbent assay
SOFA: Sequential organ failure assessment
LOS: Length of stay
ICU: Intensive care unit
IQR: Interquartile range
SD: Standard deviation
ROC: Receiver operating characteristic
AUC: Area under the curve
LR: Likelihood ratio
YI: Youden’s index
