# Supplementary material for: Human neutrophil lipocalin, procalcitonin, c-reactive protein, and leucocyte count for prediction of bacterial sepsis in emergency department patients
Source: Scand J Trauma Resusc Emerg Med. 2025 Jul 1;33:112. doi: 10.1186/s13049-025-01429-9 (PMC12210756; doi:10.1186/s13049-025-01429-9)
Supplement: Supplementary file 1 — Supplementary Material 1 [file 13049_2025_1429_MOESM1_ESM.docx]

**Supplementary material**

**Figure S1.** ROC analysis of a model including CRP + leucocyte count + NEWS + SOFA, and individual biomarkers to predict bacterial sepsis


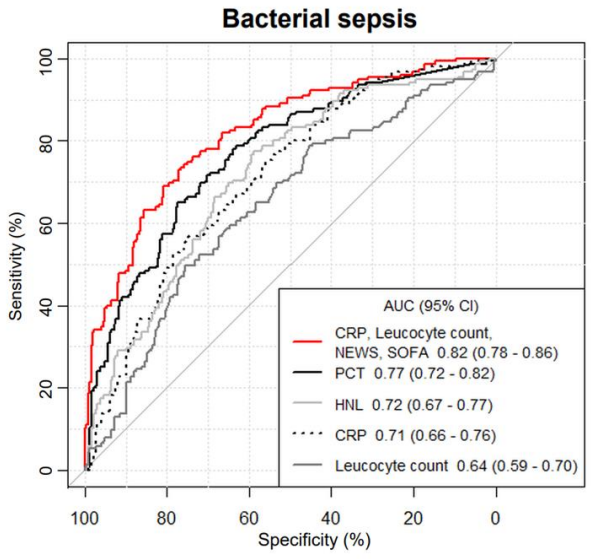


*ROC = receiver operating characteristic; AUC = area under the curve; CI = confidence interval; PCT = Procalcitonin; CRP = C-reactive protein; HNL = Human Neutrophil Lipocalin.*

**Figure S2.** ROC analysis of PCT, HNL, CRP and leucocyte count to predict bacterial infection


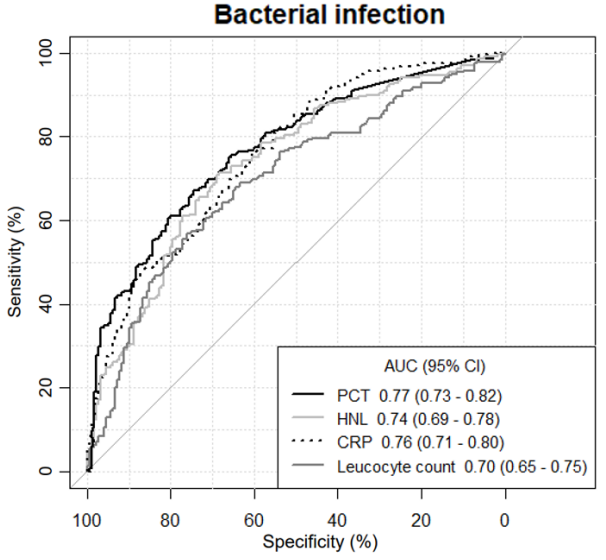


*ROC = receiver operating characteristic; AUC = area under the curve; CI = confidence interval; PCT = Procalcitonin; CRP = C-reactive protein; HNL = Human Neutrophil Lipocalin.*

**Table S1.** Performance of biomarker cut-offs to identify bacterial infection (regardless of organ dysfunction)

| Biomarker | Cut-off | Sensitivity,  % (95%CI) | Specificity,  % (95%CI) | +LR (95%CI) | -LR (95%CI) |
| --- | --- | --- | --- | --- | --- |
| CRP | 10 mg/l | 95.9 (94.0-97.8) | 28.9 (24.6-33.2) | 1.35 (1.16-1.56) | 0.14 (0.09-0.23) |
|  | 37 mg/l (YI) | 75.5 (70.9-79.6) | 61.1 (55.6-66.3) | 1.94 (1.54-2.45) | 0.40 (0.31-0.52) |
|  | 50 mg/l | 70.1 (65.6-74.6) | 65.0 (59.7-70.3) | 2.00 (1.60-2.50) | 0.46 (0.35-0.61) |
|  | 100 mg/l | 51.0 (46.3-55.7) | 81.7 (77.8-85.1) | 0.63 (0.50-0.80) | 0.60 (0.53-0.68) |
| PCT | 0.1 ng/ml | 80.1 (75.9-83.6) | 57.2 (52.0-62.3) | 1.87 (1.48-2.36) | 0.35 (0.27-0.46) |
|  | 0.16 ng/ml (YI) | 67.2 (62.5-71.6) | 74.4 (69.6-78.6) | 2.63 (2.09-3.31) | 0.44 (0.35-0.55) |
|  | 0.25 ng/ml | 55.6 (50.9-60.1) | 83.3 (79.7-86.4) | 3.33 (2.97-4.16) | 0.53 (0.45-0.63) |
|  | 0.5 ng/ml | 47.1 (42.4-51.9) | 87.2 (83.9-89.9) | 3.72 (2.93-4.72) | 0.61 (0.52-0.71) |
| HNL | 184 µg/ml (YI) | 71.0 (66.3-75.1) | 68.9 (63.7-73.6) | 2.28 (1.81-2.87) | 0.42 (0.34-0.53) |
| Leucocyte count | 10 x10^9^/l | 68.5 (63.8-72.7) | 63.3 (58.1-68.2) | 1.43 (1.15-1.78) | 0.61 (0.50-0.74) |
|  | 11.5 x10^9^/l (YI) | 56.8 (52.0-61.5) | 76.1 (71.6-80.1) | 2.38 (1.90-2.98) | 0.57 (0.47-0.69) |
|  | 15 x10^9^/l | 30.7 (26.5-35.2) | 90.6 (87.8-92.8) | 1.72 (1.34-2.21) | 0.85 (0.78-0.93) |

*CRP = C-reactive protein; PCT = Procalcitonin; HNL = Human Neutrophil Lipocalin; YI = Youden’s Index*
